# Role of Host Volatiles in Regulating the Rhythmic Host Alternation in the Mulberry Longhorn Beetle, *Apriona germari*

**DOI:** 10.3390/insects17050448

**Published:** 2026-04-24

**Authors:** Wenbo Wang, Yang Yang, Yangyixue Feng, Min Xiao, Tian Xu

**Affiliations:** Co-Innovation Center for the Sustainable Forestry in Southern China, Nanjing Forestry University, Nanjing 210037, China; wbwang@njfu.edu.cn (W.W.); yyang@njfu.edu.cn (Y.Y.); fyyx0302@163.com (Y.F.); xiaomin10313@163.com (M.X.)

**Keywords:** *Apriona germari*, wood-boring pests, diel rhythm, host alternation, aggregation behavior, host volatiles

## Abstract

Host alternation is a common behavioral strategy among many herbivorous insects. The mulberry longhorn beetle, *Apriona germari* is a destructive wood-boring pest. The adults exhibit rhythmic host alternation between feeding and oviposition hosts. However, the temporal rhythm and regulatory mechanisms underlying this behavior remain unclear. In the present study, by observing the dynamics of the numbers of *A. germari* on mulberry (*Morus alba*, feeding host) and willow (*Salix babylonica*, oviposition host) trees in a cage, we first found that both females and males began to aggregate on mulberry trees at dawn. Following 18:00 at dusk, the number of females on mulberry declined sharply, whereas the number of males decreased slightly, with a greater proportion of males staying on mulberry. To investigate the role of host volatiles in regulating the host alternation in *A. germari*, we then carried out two-choice olfactory assays to test whether the behavioral responses of *A. germari* to the host volatiles from mulberry and willow twigs differed between 04:00 and 08:00 (dawn, aggregated on mulberry) and 18:00 to 22:00 (dusk, dispersed from mulberry). Males were consistently attracted to the volatiles from healthy mulberry at both dawn and dusk, but not to those from willow. Females showed no attraction to the volatiles from either of two hosts at dawn; however, at dusk, they were significantly attracted to willow volatiles. Furthermore, volatiles from feeding-damaged mulberry trees were repellent to both sexes, particularly to males at dawn. These results indicate that the rhythmic host alternation in *A. germari* is partly mediated by host volatiles and an unidentified male-produced pheromone may be present in this species. Our findings can deepen the understanding of the host alternation in longhorn beetles, and offer a theoretical foundation for developing semiochemical-based, eco-friendly strategies for controlling this pest.

## Simple Summary

The mulberry longhorn beetle, *Apriona germari*, is a destructive wood-boring pest that causes significant economic and ecological damage to multiple hardwood species across its extensive distribution range. Adults of this species exhibit a distinct diel rhythm of host alternation, aggregating on feeding hosts (*Morus alba*) during daytime and dispersing to oviposition hosts (*Salix babylonica*) at night. However, the regulatory mechanisms underlying this rhythmic behavior remain poorly understood. In this study, we investigated the role of host volatile organic compounds (VOCs) in mediating this behavioral rhythm through cage experiments and two-choice olfactory bioassays. Our results demonstrate that male *A. germari* are consistently attracted to VOCs from undamaged mulberry throughout both dawn and dusk periods, whereas females exhibit significant attraction exclusively to willow VOCs at dusk, coinciding with their dispersal for oviposition. Notably, VOCs from feeding-damaged mulberry exhibited repellent effects, particularly toward males at dawn. These findings suggest that the rhythmic host alternation in *A. germari* is partially mediated by differential behavioral responses to host volatiles, with putative male-produced aggregation pheromones potentially contributing to the dawn reassembly on feeding hosts. This research advances our understanding of host alternation mechanisms in *Cerambycidae* and provides a theoretical foundation for developing semiochemical-based management strategies against this economically important pest.

## 1. Introduction

Herbivorous insects have evolved diverse strategies to secure the nutrients required for their physiological development and reproduction from host plants [1]. Host alternation represents a key strategy in this context [2]. Butterflies are a typical example: the larvae feed on relatively protein-rich foliage, while adults primarily obtain nutrients from nectar or sap from decaying fruits. To sustain the energy required for growth and reproduction, they are thus compelled to shift between adult-feeding hosts and oviposition hosts across different life stages [3,4,5,6,7]. Longhorn beetles (*Coleoptera*, *Cerambycidae*) are a group of xylophagous insects, which also exhibit such host alternation between adult-feeding and ovipostion hosts [8,9]. For instance, the Asian longhorn beetle, *Anoplophora glabripennis*, oviposit on *Fraxinus pennsylvanica*, while the adults do not feed on it; the adults feed on *Platanus orientalis*, but do not lay eggs on this tree [10]. The underlying mechanisms regulating the host alternation in longhorn beetles is not well understood, but this behavior makes difficulties for pest control [10,11].

The mulberry longhorn beetle, *Apriona germari* (subfamily: *Lamiinae*) is distributed in China, Japan, North Korea, Vietnam, Laos, Cambodia, Myanmar, Thailand, India, and Bangladesh [12]. In China, *A. germari* is widely distributed across 25 provinces, municipalities, and autonomous regions with the exception of Heilongjiang, Inner Mongolia, Jilin, Xinjiang, Ningxia, and Tibet [13]. Larval boring within trunks and major branches impairs host height, diameter, and volumetric growth, reduces timber quality and can lead to tree mortality under severe infestation [14]. This species primarily attacks a wide range of hardwood hosts, including *Populus* spp., *Morus alba*, *Broussonetia papyrifera*, *Ulmus pumila*, *Malus pumila*, *Cerasus pseudocerasus*, *Pyrus* spp., *Citrus reticulata*, *Salix babylonica*, etc. [15,16]. In China, *A. germari* has been listed as a “second level” harmful forest pest, with an infested area exceeding 65,000 km^2^, threatening ecological stability and economic benefits [17].

**Figure 1:**
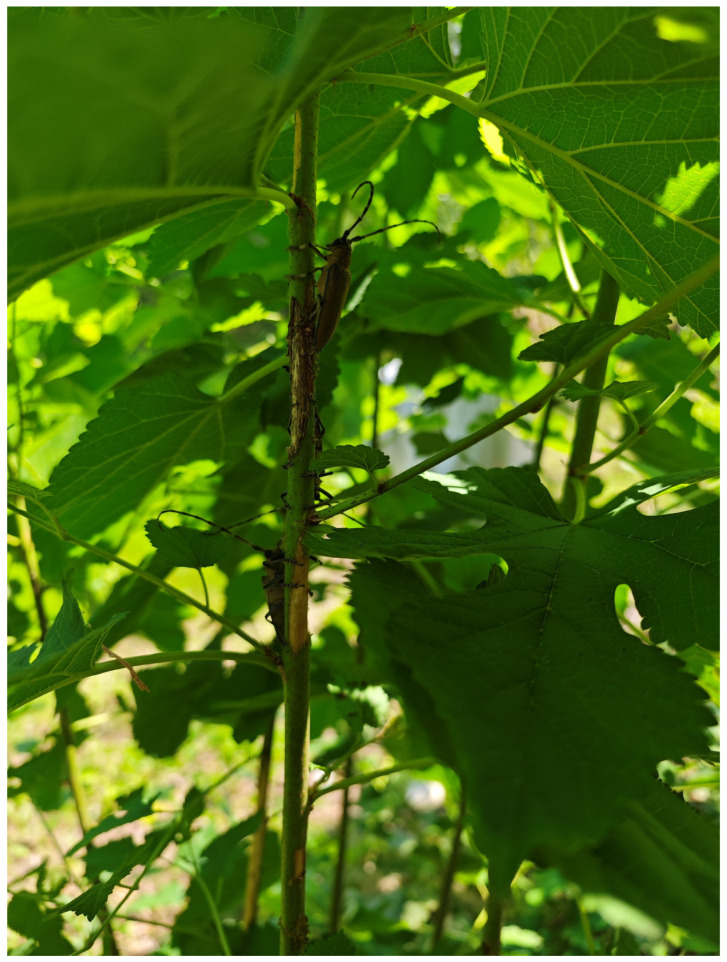
Adult *A. germari* aggregated on the twigs of *Broussonetia papyrifera*, feeding on the phloem and cambial tissues.

*Apriona germari* exhibits an extreme example of host alternation in *Cerambycidae*. Following exclusion, the adults engage in nutrient acquisition by feeding on the twigs of certain host species, including mulberry (*M. alba*) or paper mulberry (*B. papyrifera*) trees, for sexual maturation. After mating, the beetles can lay eggs on a wider range of host trees, such as polar (*Populus*), weeping willow (*Salix babylonica*), fig tree (*Ficus carica*), Planetree (*Platanus* spp.), Black locust (*Robinia pseudoacacia*) and Peking willow (*Salix matsudana*), with the exception of mulberry and paper mulberry [18,19,20], demonstrating an obligate host alternation [16,21,22]. This behavioral strategy occurs mainly because feeding on certain hosts is essential for ovarian development and production of mature eggs in females [14]. Moreover, the sexually mature adults also perform host alternation in a diel rhythm pattern. The adults feed on mulberry or paper mulberry in the daytime (Figure 1) and oviposit on different species at night [23]. However, the mechanisms underlying this host alternation remain unclear.

Host volatiles are key signals employed by longhorn beetles for host selection and location [24,25,26]. Thus, we hypothesized that the rhythmic host alternation in *A. germari* is mediated by differing responses of the beetles to odors from host trees according to the time of day. To test this hypothesis, in the present study, we first carried out a cage experiment to figure out the specific occurrence periods of host alternation between feeding and oviposition hosts (from feeding host to oviposition host, and from oviposition host to feeding host) within a day. We then investigated whether *A. germari* exhibited different preferences toward the volatiles emitted by feeding and oviposition hosts during the occurrence periods of host alternation.

## 2. Materials and Methods

### 2.1. Sources of Insects and Plants

**Figure 2:**
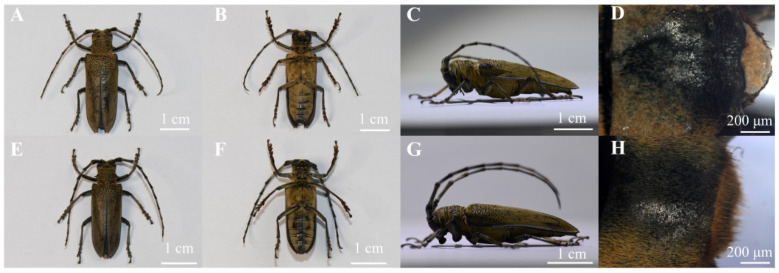
Photographs illustrating the morphological differences between male and female *A. germari*: dorsal, ventral, lateral views, and terminal abdominal segments (photographed by Wenbo Wang). Dorsal view of female (**A**) and male (**E**) *A. germari*. Ventral view of female (**B**) and male (**F**) *A. germari*. Lateral view of female (**C**) and male (**G**) *A. germari*. The abdominal tip of female (**D**) and male (**H**) *A. germari*.

All *A. germari* adults were collected from *M. alba* and *B. papyrifera* in Zhanglou Bridge, Xuzhou, Jiangsu Province, China (117°37′ E, 34°5′ N) in July 2024. The adults are 34–46 mm in length (Figure 2A,E). The integument, including the elytra, is predominantly black, overlain with a dense vestiture of fulvous, short setae, with both sexes possessing small black tubercles at the base part of the elytra (Figure 2A–G) [16]. The differences in the antennal length and body size between sexes are not significant and thus cannot serve as reliable characteristics for sex determination. However, the abdominal tip of the male is covered with fulvous short setae (Figure 2H), whereas that of the female is smooth (Figure 2D). All captured beetles were individually kept in 500 mL plastic cups in the laboratory of Nanjing Forestry University (approximately 25 °C, 40–50% RH, 14 h light and 10 h darkness). Three to four *B. papyrifera* twigs (about 3–4 cm in length) were supplied as food for each beetle, and refreshed every 2–3 day. Host branches were obtained at the campus of Nanjing Forestry University. Saplings of willow *S. babylonica* and mulberry *M. alba* (~1.2 m in height, ~3 cm in trunk diameter) were used in the cage experiment and olfactory assays. The saplings were watered daily.

### 2.2. Cage Experiment

**Figure 3:**
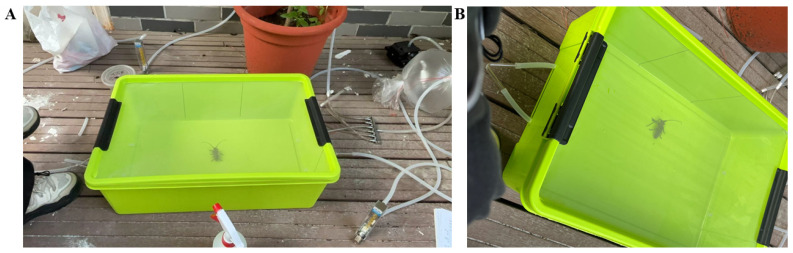
Photographs of the experimental setup used in olfactory assays. (**A**) Top view; (**B**) Side view.

A nylon cage (3 m × 3 m × 3 m) was divided into 9 regions (1 m × 1 m × 3 m each) (Figure 3A). The top of the cage was covered with black sun shade net. Two mulberry saplings (designated as M1, M2) and two willow saplings (designated as W1, W2) were placed respectively at each of the four corner areas (Figure 3B). The experiments were carried out twice on 7 July and 23 July 2024, respectively, in three cages on a vacant land in Xiashu forestry station of Nanjing Forestry University, Zhenjiang, Jangsu Province, China (119°23′ E, 32°12′ N). The beetles (4 females and 4 males, starved for 24 h) were released in the central area of each cage at 14:00 and the experiment lasted for 72 h. Every 2 h the numbers of males and females on each sapling were recorded, with each cage taking approximately 3–5 min to observe. Additionally, the numbers of oviposition pits on each sapling were also counted every 24 h. Each pot was watered following 14:00 observation. The trials initiated on 7 July were conducted under an ambient temperature of 30.4 ± 3.4 °C and a relative humidity of 67.2 ± 9.4%; the trials initiated on 23 July were conducted under an ambient temperature of 30.9 ± 2.3 °C and 76.8 ± 11.6% relative humidity.

### 2.3. Olfactory Assay

#### 2.3.1. Experiment 1

Because the cage experiment revealed that the dawn and dusk were two major periods for host alternation in *A.germari* within a day, outdoor olfactory assays were then carried out at 04:00–08:00 (dawn) and 18:00–22:00 (dusk). The experimental apparatus was a plastic storage box (Length:60 cm × Width:40 cm × Height:18 cm) (Figure 3). Two small holes were made at each end to allow airflow to enter the setup. The central opening refers to the lid on top of the box, which was cut open and covered with nylon mesh. This design allowed for air exhaust while also facilitating observation. The experimental setup was colored green to mimic the natural foliage environment where *A. germari* adults typically live, which was intended to reduce stress and promote more natural behavioral responses during the assays. A branch of a sapling, containing approximately four to five leaves, was enclosed with a polyethylene bag. Carbon-filtered air was pumped into the bag by an air pump (QC-1B, Beijing Kean Labor Protection New Technology Co., Beijing, China), which was then directed to one short side of a green-colored plastic container at a constant flow rate of 1 L/min. The other short side of the container was supplied with clean air, which had also been carbon-filtered, at the same flow rate. The plastic container was evenly divided into three sections: left, central, and right. The black lines on the inner wall of the container shown in Figure 3 indicate the artificial demarcation marks. A single beetle (female or male) was subsequently released at the center of the container. The residence time of each beetle on either side of the arena was recorded over a 15 min observation period. The trials were conducted under an ambient temperature of 31.4 ± 0.68 °C and a relative humidity of 56.3 ± 7.9%. Since adult *A. germari* feed mainly during the daytime but not at night [23], the beetles tested at dawn were starved for 12 h, whereas those tested at dusk were not starved. The container was cleaned with ethanol after testing each beetle. The beetles were tested once per day, but none were re-tested with the same stimuli. The preferences of beetles to the following odors were tested: *M. alba* vs. air, and *S. babylonica* vs. air. The streams of plant volatiles and air were physically alternated between two trials to prevent potential position effects. Each treatment was tested with 16 males (*N* = 16) and 16 females (*N* = 16).

#### 2.3.2. Experiment 2

Because previous studies have found that the volatiles from feeding-damaged hosts can attract the adults in some cerambycid species [27], we then tested whether the aggregation of *A. germari* on mulberry could be attributed to the attraction of the volatiles released from the feeding-damaged host. Two pairs of female and male *A. germari* were allowed to feed on a mulberry sapling continuously for 24 h in a nylon mesh. Then, the preferences of beetles between the odors from feeding-damaged mulberry and air were tested following the same methods described in Experiment 1. All tests were also performed between 04:00 and 08:00 and 18:00–22:00 at 29.8 ± 1.1 °C and 48.3 ± 1.7% relative humidity. A total of 16 males and 16 females were tested at dawn or dusk, respectively (*N* = 16).

### 2.4. Statistical Analysis

For the cage experiment, binomial tests were used to detect whether the ratio of female to male on mulberry or willow significantly deviated from 1:1 at different time points. Paired *t*-test was used to detect the differences in the numbers of oviposition pits on mulberry and willow. Since the data from the olfactory assays failed to follow normal distribution (Shapiro–Wilk test), Wilcoxon signed rank test was used to analyze the differences in the residence time of beetles staying in different areas. All analyses were performed using Microsoft Excel (Microsoft office 365. Released 2017. Redmond, WA, USA) and SPSS 21 (IBM Corp. Released 2012. IBM SPSS Statistics for windows, Version 21.0. Armonk, NY, USA).

## 3. Results

### 3.1. Cage Experiment

**Figure 4:**
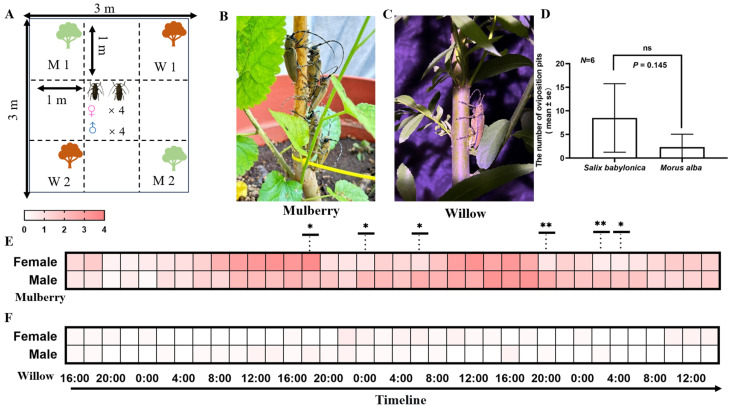
The daily host alternation of adult *A. germari* between mulberry and willow trees in the cage and the numbers of oviposition pits. (**A**) Plan view of the experimental setup, subdivided into nine 1 m × 1 m × 1 m sections. Two mulberry and two willow saplings were placed at four corners, denoted by M and W, respectively. Four females and males were released in the central area. (**B**) In the daytime, multiple *A. germari* were aggregating on a mulberry sapling for feeding and copulation (Photo credit: Tian Xu). (**C**) At night, a female *A. germari* was ovipositing on a willow sapling (Photo credit: Tian Xu). (**D**) The numbers of oviposition pits on two mulberry and willow saplings in a cage after 72 h. (**E**) The mean numbers of male and female *A. germari* on mulberry at different time points during 72 h (the specific values of the mean and standard error in each cell are shown in Table S1). (**F**) The mean numbers of male and female on willow at different time points during 72 h (the specific values of the mean and standard error in each cell are shown in Table S2). In (**D**), the date are shown as mean ± se. In (**E**,**F**), the data in the heat map are shown as the mean, each cell represents the abundance of beetles on either mulberry or willow at the corresponding time point, with darker shades indicating greater numbers, and lighter shades indicating smaller numbers. In (**E**), asterisks denote a statistically significant deviation from a 1:1 sex ratio on mulberry (Binomial test: * 0.01 < *p* < 0.05, ** 0.001 ≤ *p* ≤ 0.01). ns indicates no significant.

The diel rhythms of host alternation were generally similar between two sexes in *A. germari*. The numbers of females and males on mulberry both increased mainly from 04:00 to 08:00. In both sexes, the first peak appeared at 10:00 followed with a relatively stable number until 18:00. During the first 24 h period (16:00–16:00, +1 day), there was no significant difference between the numbers of females and males on mulberry until 18:00 (Binomial test, *p* = 0.044). The numbers of both sexes on mulberry decreased after 18:00. Compared to that of males, the number of females showed a faster rate of decline in the first two hours after 18:00 (Female: 72%; Male: 10%). During 20:00 (+1 day)–08:00 (+2 day), males were more abundant than females on mulberry, with significant differences detected at 00:00 and 06:00 (Binomial test, 00:00: *p*= 0.016; 06:00: *p* = 0.011). At the following time points, the numbers of both sexes on mulberry were consistent with the aforementioned temporal pattern (Figure 4E). On willow, the numbers of both sexes were low at each time point; no significant difference was detected between the numbers of sexes at all time points (Figure 4F).

Oviposition pits were found on both mulberry and willow. The number of oviposition pits on the two willow saplings in a cage (~8.5 in average) was greater than that on the two mulberry saplings (~2.3 in average), but the difference was not statistically significant (Paired *t*-test, *df* = 5, *p* = 0.145) (Figure 4D).

### 3.2. Olfactory Assays

#### 3.2.1. Experiment 1

**Figure 5:**
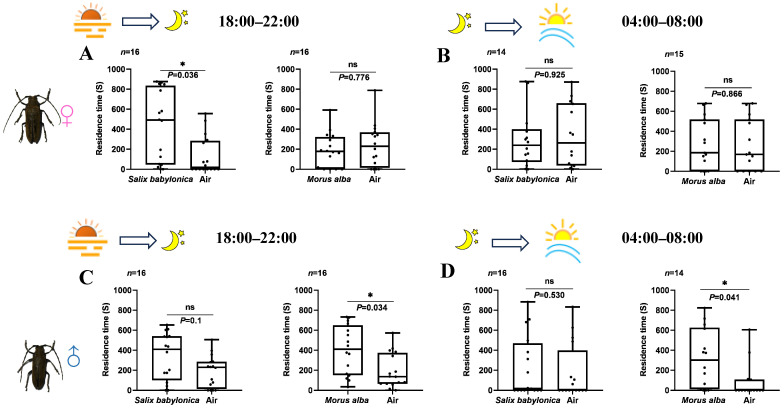
Residence time of female and male *A. germari* in the areas with plant volatiles at dusk (18:00–22:00) and dawn (04:00–08:00). Residence time of females (*M. alba* vs. Air, *S. babylonica* vs. Air) at dusk (**A**) and dawn (**B**). Residence time of males (*M. alba* vs. Air, *S. babylonica* vs. Air) at dusk (**C**) and dawn (**D**). An asterisk indicates significant difference between the residence time of opposite stimuli (*p* < 0.05). ns indicates no significant difference between the residence time of two stimuli (*p* ≥ 0.05). All analyses were performed by using Wilcoxon signed rank test.

During the dusk (18:00–22:00), females exhibited a significantly longer residence time on the side with *S. babylonica* volatiles than on the side with clean air (Wilcoxon signed rank test, *p* = 0.036), whereas the residence time of females on the side with *M. alba* volatiles almost equaled that of the females on the side with clean air (Wilcoxon signed rank test, *p* = 0.776; Figure 5A). No significant difference was detected in the odor preference of females during the period from 04:00 to 08:00 (Wilcoxon signed rank test, *S. babylonica* vs. Air, *p* = 0.925; *M. alba* vs. Air, *p* = 0.866; Figure 5B). The residence time of males on the side with *S. babylonica* was longer than that on the side with clean air during both dusk and dawn, but the difference was not statistically significant (Wilcoxon signed rank test, dusk, *p* = 0.1; dawn, *p* = 0.53; Figure 5C,D). However, the residence time of males on the side with *M. alba* odors was significantly longer than that on the side with clean air during both dusk and dawn (Wilcoxon signed rank test, dusk, *p* = 0.034; dawn, *p* = 0.041); Figure 5C,D). Few beetles did not move during the observation at dawn (*S. babylonica* vs. Air, 2 females; *M. alba* vs. Air, 1 females and 2 males).

#### 3.2.2. Experiment 2

**Figure 6:**
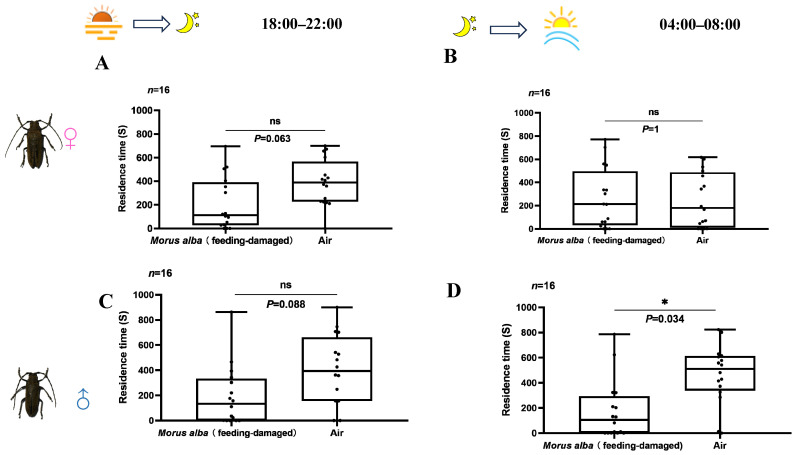
Residence time of female and male *A. germari* in the areas with the volatiles from the mulberry trees that had been fed upon by adult *A. germari* and in the areas with clean air at dusk (18:00–22:00) and dawn (04:00–08:00). Residence time of females at dusk (**A**) and dawn (**B**). Residence time of males at dusk (**C**) and dawn (**D**). An asterisk indicates significant difference between the residence time of opposite stimuli (*p* < 0.05). ns indicates no significant difference between the residence time of two stimuli (*p* ≥ 0.05). All analyses were performed by using Wilcoxon signed rank test.

From 18:00 to 22:00, both male and female were more attracted to clean air compared to the volatiles from feeding-damaged mulberry trees, and the differences were marginally significant (Wilcoxon signed rank test, Male, *p* = 0.088; Female, *p* = 0.063; Figure 6A,C). From 04:00 to 08:00, the residence time of females on the side with clean air was equal to that on the side with damaged *M. alba* volatiles (Wilcoxon signed rank test, *p* = 1; Figure 6B), while the residence time of males on the side with clean air was significantly longer than that on the side with damaged *M. alba* odors (Wilcoxon signed rank test, *p* = 0.034; Figure 6D).

## 4. Discussion

Rhythmic behaviors are widespread in insects, with most species exhibiting distinct patterns in sleep and arousal, courtship and mating, foraging, oviposition, and memory formation, while also sustaining seasonally manifested behaviors like migration and diapause [28,29,30,31]. *A. germari* exhibits rhythmic patterns in host alternation. In the present study, our results revealed that male and female adults of *A. germari* regularly aggregated on the feeding host, mulberry, from dawn to dusk, after which the majority of the beetles dispersed and did not return to mulberry until the following morning and no aggregation was observed on either mulberry or the oviposition host, willow, at night. The major periods of host alternation occurred at dawn and dusk. More oviposition pits were made on willow than mulberry but the difference was not significant. The results of olfactory assays suggest a differentiation in the responses of female and male *A. germari* to the volatiles from different kinds of hosts. Males were significantly attracted to the volatiles from undamaged mulberry, regardless of dawn or dusk, while females were only significantly attracted to the volatiles from willow at dusk. The volatiles from mulberry that had been fed upon by *A. germari* repelled the beetles, particularly the males at dawn.

While the research on insect behavioral rhythms has predominantly focused on model species, very little has been known among the longhorn beetles [28,32]. In this study, we found that the rhythms of feeding behavior in female and male *A. germari* showed a similar pattern, observed mainly in the daytime. Both sexes were observed to forage exclusively on mulberry, with no feeding activity found on willow. However, the temporal dynamics of the decline in the numbers on mulberry differed between sexes at dusk. After 18:00, the number of females on mulberry trees declined sharply, while the number of males reduced more gradually. This phenomenon may be attributed to the need for the females to locate oviposition hosts and lay eggs after completing mating and nutrient supplementation, whereas males are free from this reproductive demand. This suggests that females may prefer to select willow for oviposition. The number of oviposition pits on willow and mulberry did not differ significantly, which may be attributed to the relatively small sample size of the experiment. However, the numbers of *A. germari*, regardless of sexes, on willows were constantly low during the experiment. Previous studies have found that *A. germari* are very active at night, flying to search for mates and oviposition hosts [33]. Thus, the beetles may not stay on a host tree for a long time as in the daytime. In addition, we counted the numbers of beetles on host trees every 2 h. This time interval is much longer than the duration of making an oviposition pit (15–25 min) and laying an egg (3–5 min) by female *A. germari* [33].

Host volatiles are key signals mediating host recognition and location in longhorn beetles [34,35], and can also enhance the attraction of pheromones [36]. The results from our olfactory assays indicated that male *A. germari* were consistently attracted to the volatiles from undamaged mulberry trees during both dawn and dusk. The attraction of the odors from feeding host at dawn can lead to the aggregation of males on mulberry observed in the daytime. Nevertheless, very few males were observed staying on mulberry at night, which may be due to the high movement activity at night and the relatively long observation intervals in the experiment. The females exhibited significant preference to the odors from willow at dusk, which is consistent with the preference of ovipostion host and the oviposition behavior only performed at night.

Unexpectedly, our results suggested that female *A. germari* were not significantly attracted to the volatiles from undamaged mulberry at either dawn or dusk, even they were observed aggregating on mulberry in the daytime. Previous studies have reported that the volatiles from damaged hosts, including both mechanically and feeding damaged hosts, were attractive to some cerambycid species such as *A. glabripennis* and *Anoplophora chinensis* [34,37]. Therefore, we then tested whether the odors from feeding-damaged mulberry can attract the beetles, especially the females at dawn. However, neither male nor female *A. germari* were attracted to volatiles from feeding-damaged mulberry. On contrary, the volatiles from feeding-damaged mulberry exhibited a repellent effect on the beetles, particularly the males at dawn. Thus, the volatiles released from feeding-damaged mulberry are not the signals orienting female *A. germari* back to mulberry trees at dawn. The damages of herbivorous insects can induce the hosts to produce specific volatiles or change the composition of volatiles [38], which can repel herbivorous insects as a defensive strategy in plants [39]. For the herbivorous insects, the repellent function of the odors from damaged hosts may be able to help reduce the competition of mates and food sources [40,41].

Combining the results from the cage experiment and olfactory assays, we hypothesize that males of *A. germari* may produce an aggregation-sex pheromone or sex pheromone. Males are attracted to mulberry volatiles, and the pheromone they emit may mediate the locations of mates/conspecifics and feeding hosts in females or both sexes. In the subfamily *Lamiinae*, pheromones are typically produced by males and are attractive to both sexes (as aggregation-sex pheromone) [34,42]. Moreover, we suppose that this pheromone may be emitted at dawn, because most of beetles returned back to mulberry during this period. In *Lamiinae*, some species, such as *A. glabripennis*, *A. chinensis* and *Monochamus* spp., can rhythmically release aggregation pheromones within a day [43], which seems also exist in *A. germari*. This phenomenon differs from that observed in aphids and some other insects, which is driven by photoperiod and host plant phenology [44]. Instead, the host alternation in *A. germari* may be co-regulated by the circadian rhythms of adult activity and the timing of pheromone release. Further investigations are strongly recommended to identify the potential pheromone component in *A. germari* and characterize its temporal release patterns, which, combined with the identification of host volatiles from mulberry or willow, can contribute to developing semiochemical-based eco-friendly pest management tools.

## Data Availability

The original contributions presented in this study are included in the article/Supplementary Material. Further inquiries can be directed to the corresponding author.

## Supplementary Materials

The following supporting information can be downloaded at: https://www.mdpi.com/article/10.3390/insects17050448/s1, Table S1: The numbers of male and female *Apriona germari* on mulberry trees (mean ± se, *N* = 6); Table S2: The number of male and female *Apriona germari* on willow trees (mean ± se, *N* = 6).

## Abbreviations

The following abbreviations are used in this manuscript:

VOCs	volatile organic compounds
