# Supplementary material for: Role of Host Volatiles in Regulating the Rhythmic Host Alternation in the Mulberry Longhorn Beetle, Apriona germari
Source: Insects. 2026 Apr 24;17(5):448. doi: 10.3390/insects17050448 (PMC13207432; doi:10.3390/insects17050448)
Supplement: Supplementary file 1 [file insects-17-00448-s001.zip › insects-4196567-supplementary.pdf]

Table S1. The numbers of male and female *Apriona germari* on mulberry trees (mean ± se, N = 6).

Mulberry								
**Time**	**Male**	**Female**	**Time**	**Male**	**Female**	**Time**	**Male**	**Female**
16:00	0.8 ± 0.837	1.2 ± 1.304	$16{:}00^{+1}$	2.4 ± 1.14	3.2 ± 0.837	$16{:}00^{+2}$	3.2 ± 0.837	3.2 ± 0.837
18:00	0.8 ± 0.837	1.6 ± 1.517	$18{:}00^{+1}$	2 ± 1.225	3.6 ± 0.548	$18{:}00^{+2}$	3.4 ± 0.548	2.6 ± 1.14
20:00	0.2 ± 0.447	0.4 ± 0.894	$20{:}00^{+1}$	1.8 ± 1.483	1 ± 0.707	$20{:}00^{+2}$	2.6 ± 0.894	0.8 ± 0.837
22:00	0.6 ± 0.548	0.4 ± 0.548	$22{:}00^{+1}$	1.2 ± 0.837	0.8 ± 0.447	$22{:}00^{+2}$	2 ± 1.225	1.4 ± 1.14
00:00	0.2 ± 0.447	0.6 ± 0.548	$00{:}00^{+1}$	2.2 ± 0.837	0.8 ± 0.447	$00{:}00^{+2}$	1.8 ± 1.483	1.2 ± 1.095
02:00	1 ± 0.707	0.8 ± 0.837	$02{:}00^{+1}$	2.2 ± 0.447	1.4 ± 1.14	$02{:}00^{+2}$	2 ± 0.707	0.6 ± 0.548
04:00	0.8 ± 0.837	1 ± 1	$04{:}00^{+1}$	1.8 ± 0.447	1.2 ± 1.304	$04{:}00^{+2}$	1.8 ± 1.095	0.6 ± 0.548
06:00	1.4 ± 1.14	1.4 ± 1.342	$06{:}00^{+1}$	2.6 ± 0.894	1 ± 1.225	$06{:}00^{+2}$	1.8 ± 1.483	0.8 ± 0.447
08:00	1.2 ± 0.837	2.4 ± 1.342	$08{:}00^{+1}$	2.4 ± 1.14	1.8 ± 0.837	$08{:}00^{+2}$	1.2 ± 1.095	1.2 ± 0.447
10:00	2.2 ± 0.837	3 ± 1.225	$10{:}00^{+1}$	2.8 ± 0.837	3 ± 1	$10{:}00^{+2}$	1.2 ± 0.837	1.6 ± 1.342
12:00	2.2 ± 1.483	3.2 ± 0.837	$12{:}00^{+1}$	2.6 ± 1.14	3.4 ± 0.548	$12{:}00^{+2}$	1.4 ± 0.894	1.6 ± 1.342
14:00	2 ± 1.414	3.4 ± 0.548	$14{:}00^{+1}$	3.4 ± 0.548	2.8 ± 1.095	$14{:}00^{+2}$	1.6 ± 0.548	1.4 ± 1.517

The superscript on the time indicates the number of days of the experiment.

Table S2. The number of male and female *Apriona germari* on willow trees (mean ± se, *N* = 6).

Willow								
**Time**	**Male**	**Female**	**Time**	**Male**	**Female**	**Time**	**Male**	**Female**
16:00	0.2 ± 0.447	0.2 ± 0.447	$16:00^{+1}$	0.2 ± 0.447	0.2 ± 0.447	$16:00^{+2}$	0.4 ± 0.548	0
18:00	0.2 ± 0.447	0.2 ± 0.447	$18:00^{+1}$	0.4 ± 0.548	0	$18:00^{+2}$	0	0.2 ± 0.447
20:00	0	0.2 ± 0.447	$20:00^{+1}$	0.2 ± 0.447	0	$20:00^{+2}$	0.2 ± 0.447	0
22:00	0	0	$22:00^{+1}$	0.4 ± 0.548	0.6 ± 0.548	$22:00^{+2}$	0	0
00:00	0	0.2 ± 0.447	$00:00^{+1}$	0.2 ± 0.447	0.4 ± 0.548	$00:00^{+2}$	0	0.2 ± 0.447
02:00	0.2 ± 0.447	0	$02:00^{+1}$	0.2 ± 0.447	0.2 ± 0.447	$02:00^{+2}$	0.2 ± 0.447	0
04:00	0	0	$04:00^{+1}$	0.2 ± 0.447	0.4 ± 0.548	$04:00^{+2}$	0	0
06:00	0.2 ± 0.447	0	$06:00^{+1}$	0	0.4 ± 0.548	$06:00^{+2}$	0	0
08:00	0.2 ± 0.447	0	$08:00^{+1}$	0.2 ± 0.447	0.2 ± 0.447	$08:00^{+2}$	0	0
10:00	0.2 ± 0.447	0	$10:00^{+1}$	0	0.4 ± 0.548	$10:00^{+2}$	0	0.4 ± 0.548
12:00	0.4 ± 0.548	0	$12:00^{+1}$	0	0.2 ± 0.447	$12:00^{+2}$	0	0.4 ± 0.548
14:00	0.4 ± 0.548	0	$14:00^{+1}$	0	0.2 ± 0.447	$14:00^{+2}$	0	0.4 ± 0.548

The superscript on the time indicates the number of days of the experiment.
